# Rapid increase of syphilis in Tokyo: an analysis of infectious disease surveillance data from 2007 to 2016

**DOI:** 10.5365/wpsar.2017.8.2.006

**Published:** 2019-03-27

**Authors:** Yoshiyuki Sugishita, Aya Kayebeta, Kumiko Soejima, Mariko Yauchi

**Affiliations:** a Infectious Disease Control Section, Health and Safety Division, Bureau of Social Welfare and Public Health, Tokyo Metropolitan Government.

## Abstract

The objective of this study was to examine the trends of primary and secondary syphilis in Tokyo between 2007 and 2016 using national infectious disease surveillance data. We analysed all 3269 cases reported during these 10 years. A statistically significant increase in cases was observed after 2010 with a more rapid rate of increase after 2014 mainly in urban areas in Tokyo. The notification rates per 100 000 population in 2010, 2014 and 2016 were 0.9 (*n* = 113), 2.2 (*n* = 295) and 8.7 (*n* = 1190), respectively. Domestic syphilis transmission was suspected in 92.6–99.3% of cases during the period 2007–2016.

Until 2013, the increase was mainly observed among men who have sex with men (MSM); however, heterosexual transmission became more dominant and eventually surpassed transmission among MSM in 2015. In 2016, the notified cases of infections through heterosexual contact were 22.3 and 40.4 times higher in men and women, respectively, compared to those in 2010. The median ages of affected heterosexual men and women were 37 (interquartile range: 28–46) and 26 (interquartile range: 22–32) years, respectively. Reports of oropharyngeal lesions have been increasing among both men and women with syphilis. The number of congenital syphilis cases reported in Tokyo was 0 to 3 cases per year during the study period.

More information and further analysis are needed to explain the reason for this increase.

## Introduction

Syphilis is a common sexually transmitted infection. In 2012, an estimated 5.6 million new syphilis infections among people aged 15 to 49 years were reported globally. (*1*) In Japan, a venereal disease prevention law passed in 1948 mandated a syphilis patient notification system. The Ministry of Health, Labour and Welfare consolidates data using the National Epidemiological Surveillance of Infectious Disease (NESID) system. (*2*, *3*) Although syphilis cases nationwide decreased from 216 617 in 1948 to 621 in 2010, they rebounded afterwards, reaching 4546 in 2016. (*2*, *3*)

## Methods

### Surveillance

Medical institutions report cases to NESID through public health centres. In 2015, 31 public health centres and approximately 13 600 medical institutions served the 13.5 million residents of Tokyo. (*4*, *5*) Syphilis diagnosis and treatment are widely available throughout the metropolitan area, including at community medical facilities. Free and anonymous syphilis and HIV testing is also offered by most municipal public health centres and the Tokyo Metropolitan Testing and Counselling Offices.

Physicians are required to report cases of early symptomatic (primary and secondary [P&S]) syphilis, late symptomatic syphilis, asymptomatic syphilis and congenital syphilis (CS) via facsimile to public health centres using a designated paper notification form. Public health centre staff then register cases online to NESID. Demographic and clinical information, date of diagnosis and epidemiological information (e.g. location of disease transmission, sexual history) are consolidated in NESID. All registered syphilis cases are verified by surveillance officers in the Tokyo Metropolitan Infectious Disease Surveillance Center (TMIDSC), which publishes weekly surveillance reports. (*6*)

### Data collection

**Fig. 1:**
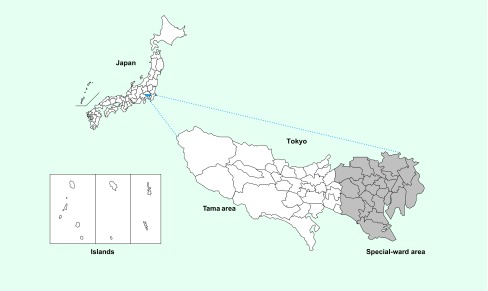
**Map of Tokyo (includes 23 special wards, Tama area, and islands)**

We extracted cases of P&S syphilis and CS in Tokyo from 1 January 2007 to 31 December 2016 from NESID. Tokyo consists of 23 special wards (central), the Tama area (suburban) and the islands (suburban) (**Fig. 1**). We used NESID surveillance definitions to define early symptomatic syphilis and CS. Early symptomatic syphilis was defined as an individual who tested positive in both nontreponemal and specific treponemal tests with at least one clinical sign or symptom (primary: painless chancre; secondary: painless inguinal lymphadenopathy, syphilitic roseola, papular syphilide or condyloma lata). (*2*, *3*) CS was defined as a live infant with signs or symptoms of CS or a positive serological examination. (*2*, *3*)

### Descriptive analysis

We performed descriptive analysis of early symptomatic syphilis cases considering sex, age, diagnosis date, syphilis stage, symptoms, sex of partner, suspected location of disease transmission (Tokyo, other prefectures in Japan, outside of Japan or unknown) and location of the reporting medical facility (central or suburban Tokyo). Sex partner preferences were categorized as men who have sex with men (MSM), men who have sex with women only (MSW), men who have sex with men and women (MSMW), women who have sex with men only (WSM), women who have sex with women (WSW), women who have sex with women and men (WSWM) or sexual contact type unknown. The notification rate was calculated to indicate the annual number of newly diagnosed and notified cases per 100 000 population using the annual population estimates from the 2010 and 2015 Census in Tokyo.

Descriptive analysis of CS was performed considering date of diagnosis.

### Regression analysis

We focused on P&S syphilis to analyse trends in cases using a generalized linear model. We offset a Poisson regression model by the estimated annual population and compared every pair of adjacent years by two-sample tests for equality of proportion. For comparison, we employed the notified cases of P&S syphilis and the estimated annual population of Tokyo.

All statistical analyses were done with R software version R-3.4.1 (R Foundation for Statistical Computing, Vienna, Austria). A *p* value of < 0.05 was considered statistically significant. We performed Bonferroni corrections for multiple comparisons. The number of tests that compared adjacent years was nine, so the significance level for each test was set to 0.0056.

### Ethics statement

This study was exempt from ethical review committee review since the data were surveillance data conducted under the provisions of Japanese law. The data collected in this study do not contain personal information.

## Results

### Notifications and rates

#### Overall and by sex

**Fig. 2:**
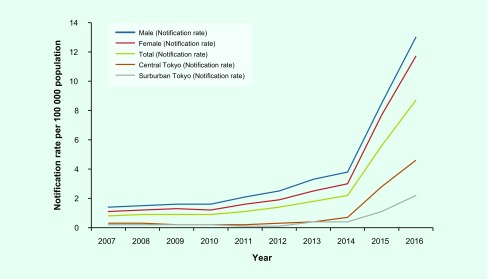
**Notification rates per 100 000 population of primary and secondary syphilis by sex and area, Tokyo, 2007 to 2016**

The notification rate of P&S syphilis was 8.7 per 100 000 population (*n* = 1190) in 2016, 9.7 times higher compared to 0.9 (*n* = 113) in 2010 and a fourfold increase compared to 2.2 (*n* = 295) in 2014 (**Fig. 2**). The annual notification rate for men exceeded that for women throughout the period of 2007 to 2016. Both men and women had the highest notification rate in 2016, which was 13.0 (*n* = 875) for men and 4.6 (*n* = 315) for women.

#### Sources of notification reports

Annual notification rates from central Tokyo exceeded those from the suburbs (**Fig. 2**). In central Tokyo, the notification rate was 1.1 per 100 000 population in 2007, increased to 3.0 in 2014 and climbed to 11.7 in 2016. In the Tokyo suburbs from 2007 to 2012, the notification rate ranged from 0.1 to 0.2, then climbed to 2.2 by 2016.

#### Trend analysis

**Fig. 3:**
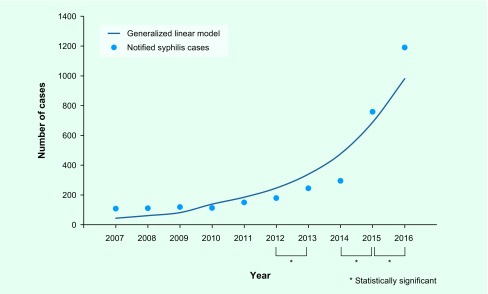
**Generalized linear model fitting and comparison of adjacent years by two-sample test for equality of proportions, by use of the number of primary and secondary syphilis notifications and estimated population of the residents in Tokyo**

The model shows that cases trended upward throughout the study period (*P* < 0.001) (**Fig. 3**). There was a statistically significant increase in the number of cases from 2012–2013, 2014–2015 and 2015–2016 (*P* < 0.0056).

### Suspected location of disease transmission

The proportions of cases with suspected transmission within Japan ranged from 92.6% to 99.3% during the study period. The proportions of domestically acquired infections stemming from Tokyo ranged from 87.6% to 96.3%.

### Disease stage

**Fig. 4:**
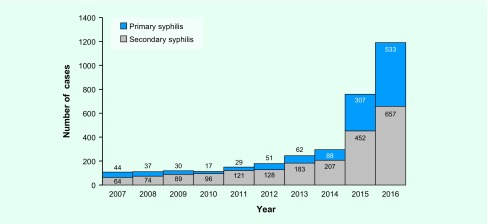
**Cases of notified syphilis by stage, Tokyo, 2007 to 2016**

A total of 1198 primary cases and 2071 secondary cases were reported from 2007 to 2016 (**Fig. 4**). The number of primary syphilis cases had been declining; they started increasing again after 2010. The number of secondary syphilis cases has consistently increased since 2007. In 2016, the number of primary cases was 533, a 31-fold increase since 2010. The number of secondary cases was 657, a sevenfold increase during the same period.

### Sex of partner

#### Men who have sex with men

**Fig. 5:**
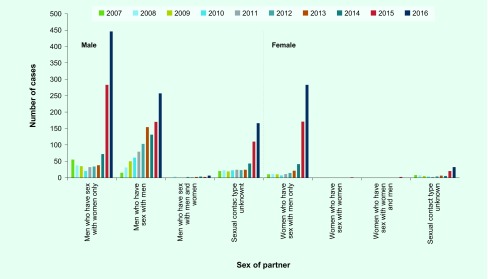
**Changes in the number of the notifications of primary and secondary syphilis cases by sex of partner, Tokyo, 2007 to 2016**

**Fig. 6:**
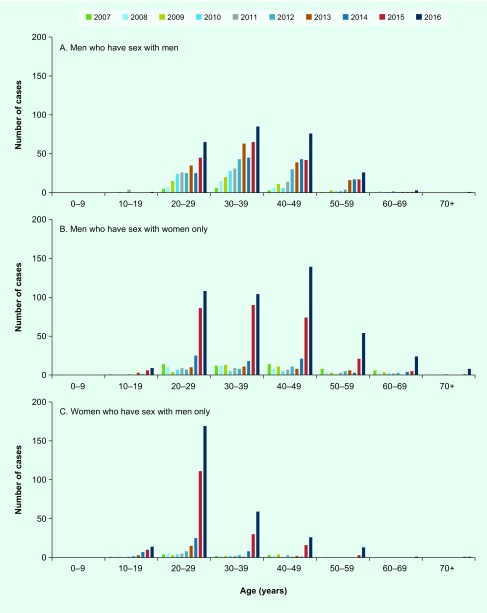
**Age distribution of primary and secondary syphilis cases among men who have sex with men, men who have sex with women only, and women who have sex with men only, Tokyo, 2007 to 2016**

**Fig. 7:**
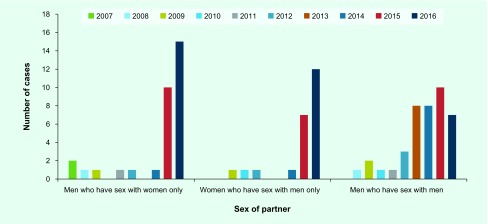
**Changes in the number of cases with oropharyngeal lesions among primary and secondary syphilis cases by sex of partner, Tokyo, 2007 to 2016**

Infections among MSM steadily increased from 2007 to 2016 (**Fig. 5**). The number of cases among MSM was 4.2 times higher in 2016 than in 2010. The proportion of MSM cases increased annually from 2007 to 2013, peaked at 62.9% in 2013 and then decreased to 21.6% in 2016. The median age of MSM during 2007–2016 was 36 (interquartile range [IQR]: 29–43) years (**Fig. 6A**). During the period 2012–2016, the incidence of oropharyngeal lesions increased (**Fig. 7**) and affected a greater percentage of MSM with syphilis (2.7% in 2016 to 6.1% in 2014.)

#### Men who have sex with women only

The number of cases among MSW decreased from 2007 to 2010, then increased in 2014, and markedly increased during the years 2015–2016 (**Fig. 5**). The cases among MSW (*n* = 283) exceeded those among MSM (*n* = 170) in 2015 and was 22.3 times higher in 2016 compared to 2010. The proportion of cases among MSW was 50.9% in 2007, decreased to 15.5–21.3% from 2010 to 2013 and increased again in 2014 to 37.5% in 2016. The median age of MSW during 2007–2016 was 37 (IQR: 28–46) years (**Fig. 6B**). Affected MSW with oropharyngeal lesions sharply increased in 2015 and 2016 (**Fig. 7**); the proportion was 3.5% in 2015 and 3.4% in 2016.

#### Men who have sex with men and women

The notified cases in MSMW were 0 to 3 cases per year in 2007 to 2015. In 2016, six cases were reported.

#### Women who have sex with men only

The notified cases in WSM were stable between 2007 and 2012; however, they increased in 2013 to 2014 and markedly increased in 2015 and 2016, mirroring the trend seen in the MSW population (**Fig. 5**). There were 40.4 times more cases in 2016 compared to 2010. The proportion of WSM cases ranged from 6.2–9.9% in 2007 to 2013; it increased in 2014, reaching 23.8% in 2016. From 2007 to 2016, the median age of affected WSM was 26 (IQR: 22–32) years; in 2016, 59.7% of the affected WSM were in their 20s (**Fig. 6C**). Oropharyngeal lesions increased among WSM in 2015 and 2016 (**Fig. 7**); the proportion was 4.1% in 2015 and 4.2% in 2016.

#### Women who have sex with women

Only one case of syphilis, reported in 2015, involved WSW.

#### Women who have sex with women and men

Two cases, reported in 2015, were in WSWM.

### Congenital syphilis

Twelve cases (0–3 cases per year) of CS were reported in Tokyo. The number of cases was one in 2008, two in 2009, three in 2012, one in 2014, two in 2015 and three in 2016.

## Discussion

We found that P&S syphilis cases in Tokyo generally increased from 2007 to 2016 and has been sharply increasing since 2015. During the period 2007–2010, an increase in infections among MSM was offset by a decrease of infections among MSW. As transmission among MSM continued to rise, the number of cases overall increased from 2011 to 2013. Cases among MSW and WSM increased rapidly after 2014, resulting in a larger number of cases transmitted through heterosexual contact than among MSM in 2015. We conclude that heterosexual transmission is a significant driver of the increase in syphilis cases in Tokyo with a contributing increase of cases among MSM.

The disproportionate increase in primary-stage syphilis may be due to increased ascertainment from prompt health-care seeking and improved recognition among clinicians. However, secondary syphilis also increased sevenfold, suggesting that the increase in primary syphilis cases may be due to increased incidence. Reports of oropharyngeal lesions increased among both men and women with syphilis. The proportion of those with oropharyngeal lesions did not vary considerably among MSM, MSW and WSM. The oral cavity can be a point of entry for syphilis, and an oropharyngeal lesion can be a source of syphilis infection. (*7*) Providers and the public should be aware that syphilis can be transmitted through oral sex.

We are concerned about the increase of syphilis in young women. An increase of syphilis among women occurred in the 2010s in the United States of America that resulted in increased CS incidence. (*8*) CS is preventable; pregnant women and their partners should be encouraged to seek appropriate prenatal care, including routine prenatal screening and treatment of syphilis.

The notification rate in central Tokyo exceeded that in the suburbs. One potential explanation is that there are more medical facilities with infectious disease departments in central Tokyo. Though the notification rate in suburban areas is low, it has increased, highlighting that syphilis is not a public health problem limited to urban areas.

During the study period, trends in syphilis cases at the national level were like those in Tokyo. National syphilis cases started increasing in 2011. Most cases were from large metropolitan areas such as Tokyo and Osaka. Cases among MSM and heterosexual men and women increased during the years 2011–2016. (*2*, *3*)

Syphilis has been increasing globally since the 2000s, (*9*) including in countries neighbouring Japan. (*10*, *11*) Global travel contributes to the spread of sexually transmitted infections; one study found that 20.4% of travellers have casual sexual contact during foreign trips. (*12*) As the Tokyo Metropolitan Government (TMG) is hosting the Olympic and Paralympic Games in 2020, it is expected that more people will visit Tokyo. Foreign visitors may introduce or acquire syphilis in Japan, potentially spreading syphilis both in Japan and their home countries.

### Limitations

We used census and surveillance data collected by government departments. Identifying the reasons behind the increased number of syphilis cases in Tokyo is beyond the scope of this study since NESID data do not contain risk factor information. Other studies outside of Japan have cited increased health-care access and utilization, improved diagnostic testing or increased high-risk sexual behaviour as reasons for increasing syphilis incidence. (*13*, *14*) Physicians may have increased reporting in response to heightened awareness that syphilis is a notifiable disease and the availability of the syphilis case reporting form on the TMIDSC web site. (*6*) Not all cases of syphilis are diagnosed; patients often do not seek medical care for an initial lesion, which is painless and disappears spontaneously. Since the clinical presentation is non-specific, syphilis testing may not be performed for those who do seek care. Some diagnosed cases are likely never reported. Also, the Prevention of Infectious Diseases and Medical Care for Infectious Patients Act has no express provision for contact tracing, (*15*) which is necessary to detect and treat sexual contacts. The number of CS cases may also be underestimated since only live births are classified as CS.

In response to the increase in syphilis cases, TMG provided clinician training sessions on testing and treatment, expanding syphilis testing opportunities, adding home address and nationality to surveillance information and updating public educational materials (including an e-learning curriculum).

Targeted interventions are needed to curb the rising number of syphilis cases. Continued surveillance and additional analysis are needed to identify and mitigate factors causing the increase. Public health strategies to prevent and treat infections in young women are imperative to preventing CS. Curbing syphilis cases in Tokyo depends on increased awareness and the collaborative efforts of health-care providers, educators, media, academia and the public.
